# Stretchable Multiresponsive Hydrogel with Actuatable, Shape Memory, and Self‐Healing Properties

**DOI:** 10.1002/advs.201800450

**Published:** 2018-06-10

**Authors:** Feng Zhang, Ligui Xiong, Yongjian Ai, Zhe Liang, Qionglin Liang

**Affiliations:** ^1^ Key Laboratory of Chemical Biology (Ministry of Education) Beijing Key Laboratory of Microanalytical Methods and Instrumentation Department of Chemistry Tsinghua University Beijing 100084 China

**Keywords:** actuation, multiresponsive hydrogels, self‐healing properties, shape memory, stretchable materials

## Abstract

Smart hydrogels with responsive behaviors have attracted tremendous attention. However, it is still a challenge to synthesize stretchable hydrogels capable of changing their original properties in response to multiple external stimuli. Here, integration of actuation function, shape memory, and self‐healing capability in a highly stretchable hydrogel under triple external triggers is achieved by rationally engineering multiple functional moieties. The hydrogel exhibits high stretchability (average relative strain (mm/mm) is >15) and excellent fatigue resistance during 100 loading cycles of 100% strain. Incorporating a moisture‐insensitive polymer film with the hydrogel, hydroactuated functionality is demonstrated. Moreover, shape memory and self‐healing abilities of the hydrogel are realized by the formation of ionic crosslinking or dynamic borate ester in conditions of multivalent cations and pH, respectively. Deformable plastic flowers are displayed in this work as a proof‐of‐concept, and it is believed that this smart hydrogel could be used in plenty of frontier fields, such as designing electronic devices, soft robotics, and actuators.

Functional materials, like actuation hydrogel,^1^ shape memory,^2^ and self‐healing polymers,^3^ have drawn increasing attentions and perform extensive applications in fields of tissue engineering,^4^ drug delivery,^5^ smart coating,^6^ and soft robotics.^7^ Up to now, efforts devoted to the development of functional materials mainly focus on combing different moieties to maximize certain type of function. Taking self‐healing hydrogels for example, combinations of dynamic covalent bonding, supramolecular interactions, and polymer chemistry have been investigated to improve healing efficiency.^8^ Even though these researches are beneficial to broaden the range of functional hydrogels, diversity of functionalities is also a key factor that deserves further investigation to improve the versatility of hydrogels. In recent years, stimuli‐responsive hydrogels,^9^ also called smart hydrogels, have been explored to meet the demand of multifunctionality, these materials are capable of adapting structure and property changes in a controlled manner to external stimuli, including moisture, pH, temperature, light, and so on. The advent of smart hydrogels significantly boosts the development of functional materials endowing hydrogels with postresponse properties upon external triggers.^10^

Many functional materials whose functionality stems from responsiveness have been reported. For example, surface modeling of polyacrylamide (PAM) hydrogel into microarrays, microstructure hydrogel showed microactuation functionality in responding to moisture changes.^11^ Bio‐inspired hydrogel generated by boronate–catechol complexation displayed excellent self‐healing performance in weak alkaline environment.^12^ And multiresponsive supramolecular hydrogel which consists of polyacrylic acid, phenylboronic acid, and agar presents shape memory functionality under triple stimuli.^13^ However, capability of current responsive hydrogels is commonly too monotonous to meet various requirements, either showing one functionality upon individual trigger,^14^ or multiresponsive behaviors all resulting in same property.^15^ Meanwhile, stretchability of hydrogel, which could be enhanced by interpenetrating network structure,^16^ acts an essential role in researches referring to soft robotics, electronic skin, and artificial muscle.^17^ Although multifunctional materials have been reported, like Harada and co‐workers^18^ engineered a supramolecular hydrogel with redox‐responsive shape memory and self‐healing abilities by employing two host–guest interactions,^19^ poor stretchability restricted the usage of such hydrogels. However, the fabrication of stretchable hydrogel with diverse properties which can respond to multiple external stimuli still remains a challenge.

Here, we described a novel multiresponsive hydrogel with actuatable, shape memory, and self‐healing properties corresponding to three types of common triggers: moisture, multivalent cations, and pH. This smart hydrogel is formed by two interpenetrating polymer chains: polyacrylamide and phenylboronic acid grafted alginate (alginate‐PBA), which is abbreviated to *i*‐PAP hydrogel. Among the gel, PAM is chosen because of its extensively studied properties and excellent compatibility with other brittle polymer networks to reinforce the final mechanical performance.^20^ Alginate, a kind of natural polysaccharides, can be rapidly cross‐linked by various multivalent cations (Ca^2+^ was used in this work) thus possessing ionic responsiveness.^21^ In particular, numerous hydrophilic groups in PAM and alginate chains greatly improve water absorption of hydrogel, then the huge volume changes caused by water content variation could act as the driving force of actuation functionality. We selected phenylboronic acid to modify alginate on account that boronic acid group is able to dynamic covalently interact with diol groups of alginate in mildly alkali conditions (pH is about 9–10)^22^ showing self‐healing behavior without destroying the original nature of alginate thoroughly.^23^ With these compatible multiple functionalities and high stretchability, this gel demonstrates promising prospects in aspects of soft robotics, electronic skin, and actuators.

**Figure** **1** illustrates synthetic process of *i*‐PAP hydrogel. The detailed procedure is provided in the Supporting Information. Pristine *i*‐PAP gel is considered as control sample in the context. The resultant gel was completely transparent with naked eyes (Figure 1b) and held a typical porous structure (Figure 1c), which differed from the lamellar structure of pure alginate‐Ca^2+^ hydrogel.^24^ Hydrogels immersed with CaCl_2_ or NaOH solution were regarded as conditioned hydrogels (Ca‐conditioned for CaCl_2_ and alkaline‐conditioned for NaOH). The formation process of conditioned gels is schematically demonstrated in Figure S1 (Supporting Information). Comparing with control gel, two kinds of conditioned samples exhibited more compact microscopic structures by scanning electron microscopy (SEM) images (Figure S2, Supporting Information). To verify differences of physiochemical properties between control and conditioned hydrogels, we carried out Fourier transform infrared spectra and rotational rheometer characterizations (Figures S3 and S4, Supporting Information).

**Figure 1:**
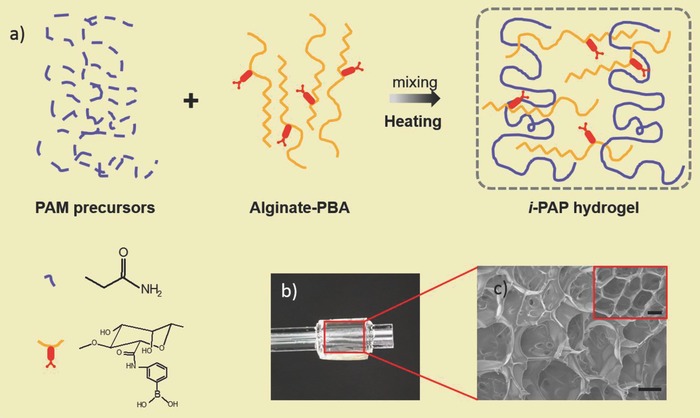
Schematic illustration of *i*‐PAP hydrogel synthetic process and microscopic structures of hydrogel. a) Schematic to detail synthetic process of *i*‐PAP hydrogel. b) Photography of hydrogel sample twisted on glass tube. c) Cross section and surface scanning electron microscopy (SEM) images of lyophilized *i*‐PAP hydrogel. Inset demonstrates the surface structure of same sample. Scale bar: 50 µm.

Owing to PAM scaffold and two interentangling polymer chains, our hybrid hydrogel demonstrated excellent stretchability (**Figure** **2**a), which is more remarkable than any single of its constituents, the polyacrylamide and alginate‐PBA gels (Figure S5, Supporting Information). Tensile ability of hydrogels was tested by a home‐made device (Figure S6, Supporting Information) and evaluated through comparing critical relative strain (defined as maximum stretching displacement without rupture to its original length (mm mm^−1^)). The average critical relative strain of gels is >15 in statistically (Figure 2b,c), with 16 for control hydrogel, 24 for Ca‐conditioned hydrogel (Figure 2b) and 20 for alkaline‐conditioned hydrogel (Figure 2c), respectively. The enhanced stretchability of hydrogels treated with Ca^2+^ or OH^−^ was probably derived from the ionic crosslinking or dynamic boronate ester bonds, thus dissipating the applied energy and enhancing the stretchability of conditioned hydrogels. To best of authors' knowledge, this is the largest stretching degree compared with the state‐of‐the‐art multistimuli‐responsive hydrogels.^13^, ^25^ Nevertheless, neither the concentration of CaCl_2_ (Figure 2b) nor immersion time of NaOH (Figure 2c) had obvious effects on the stretching performance. This result implied that bonding sites for Ca^2+^ in control gel were finite and easy to be occupied in short time. It is worth to be mentioned that immersion time of alkaline solution is limited to 1 h because polyacrylamide tends to hydrolyze under alkaline conditions.

**Figure 2:**
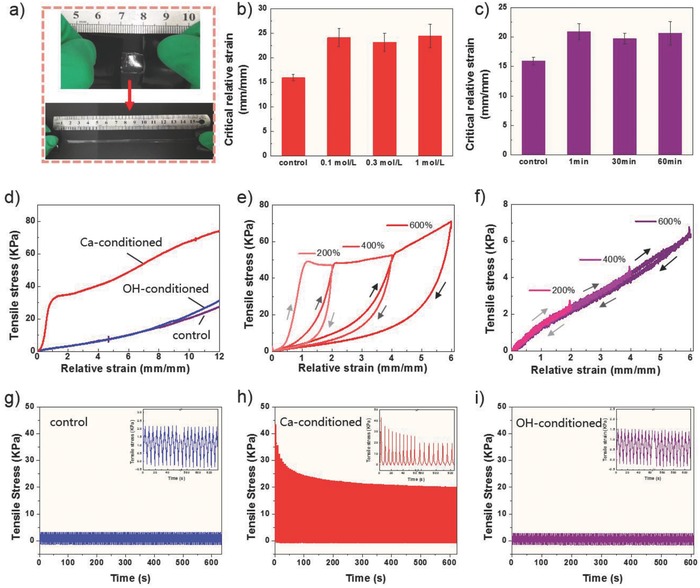
Stretching performance of original and conditioned *i*‐PAP hydrogels. a) Tensile demonstration of original *i*‐PAP hydrogel. b,c) Maximum tensile ability of *i*‐PAP hydrogel varies with concentration of CaCl_2_ and immersion time of 1 mol L^‐1^ NaOH. The results are illustrated as the mean ± s.d. of four independent experiments. d) Tensile stress–relative strain curves of three hydrogels until relative strain achieves 12. e,f) Tensile stress–relative strain curves of Ca‐conditioned hydrogel and alkaline‐conditioned hydrogel samples subjected to loading–unloading tensile cycles under 200, 400, and 600% strains, successively. g–i) Tensile stress changes with time of *i*‐PAP hydrogel, Ca‐conditioned hydrogel, and alkaline‐conditioned hydrogel samples loading with 100% strain for 100 cycles. The insets show details of corresponding curves.

To quantify tensile stresses of hydrogels, a tensile tester with a 100 N load cell was employed to monitor stretching process. As exhibited in tensile curves (Figure 2d), the larger tensile stress of Ca‐conditioned hydrogel indicated the strong ionic crosslinking interaction. Then, we loaded a sample of conditioned hydrogels up to certain tensile strains (200, 400, and 600%), unloaded the gel to zero force, and followed with a second and third loading to study recovery of hydrogels after tensile. The remarkable hysteresis loop of Ca‐conditioned hydrogel (Figure 2e) reflected the effective energy dissipation. In contrast, negligible hysteresis was detected in alkaline‐conditioned gel sample (Figure 2f). On the other hand, loading–unloading hydrogel samples in 100% strain for 100 cycles were operated. Control (Figure 2g) and alkaline‐conditioned (Figure 2i) hydrogels exhibited superior fatigue resistance^26^ as no significant tensile stress changes were observed. The reformation of hydrogen bonds between polymer chains and dynamic boronate ester bonds could account for the reinforced fatigue resistance. Maximum tensile stress of alkaline‐conditioned hydrogel (1.5 kPa) was smaller than control sample (2.0 kPa), indicating these gels were more susceptible to subtle external force (Figure 2i). Under the same conditions, tensile stress of Ca‐conditioned gel was decreased by almost 50% undergoing 100 tensile cycles (Figure 2h). Therefore, Ca‐conditioned hydrogels were unable to recover to its original state with distinct deformation after large stretching, while alkaline‐conditioned hydrogels made it at the macroscale. This could be explained that ionic crosslinking of alginate‐PBA was different from dynamic interactions and hard to reform to result in crack propagation. With predominant stretchability and fatigue resistance advantages, *i*‐PAP hydrogels had a promising potential in substrate material of wearable electronics with high sensitivity and durability.

Apart from excellent stretchability, *i*‐PAP hydrogels manifested multiple functionalities related to external stimuli. First, actuatable functionality was achieved via responsiveness to moisture. In hydration and dehydration cycle, the *i*‐PAP hydrogel showed reversible response to moisture with great volume changes. The volume of original hydrogel fiber expanded fivefolds after complete swelling in water, while returned to the original state after dehydration in air (**Figure** **3**a). To demonstrate actuation function, classical asymmetric bilayer structures^27^ were used by assembling tailored rectangular *i*‐PAP hydrogel samples with polydimethylsiloxane (PDMS) sub‐millimeter (0.5 mm) films. The PDMS films were hydrophilic modified by oxygen plasma. The Janus assembly dehydrated rapidly in 60 °C oven and bent spirally contributing to different physical properties of *i*‐PAP hydrogel and PDMS.^28^ During dehydration, contraction force generated by volume shrinkage resisted to the gravity of the whole assembly and deformed it. Deformation patterns changed with degree of dehydration until *i*‐PAP hydrogel divorced from PDMS sheet (Figure 3b). Once rehydrated, deformed assembly induced by hydrogel dehydration was able to return to its original shape, just as a simple actuator. Nevertheless, this actuation function of pure PAM gel or alginate‐PBA gel has hardly been reported to our knowledge.

**Figure 3:**
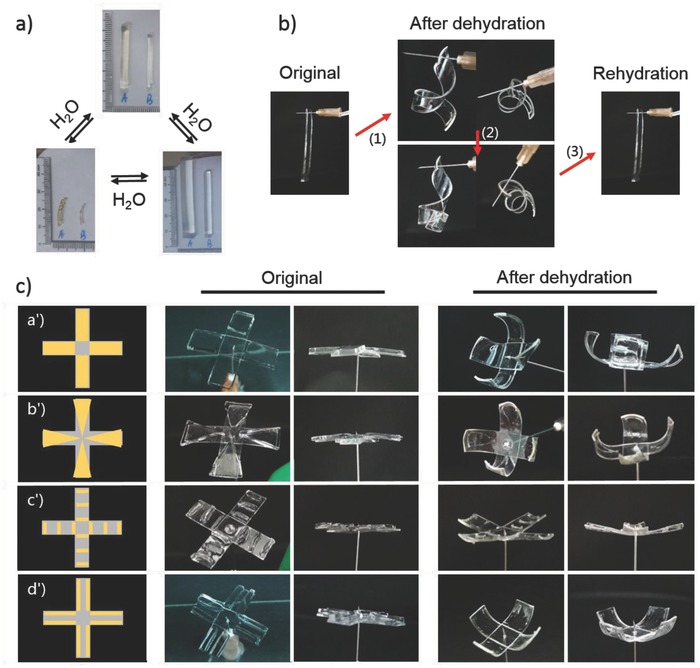
Actuatable functionality of *i*‐PAP hydrogel in response to moisture stimuli. a) Digital photograph of reversible swelling–shrinking transformation of *i*‐PAP hydrogel. b) Deformation process of Janus assembly containing hydrogel and hydrophilic PDMS during dehydration and rehydration. c) Diverse patterns of “plastic flowers.”

Not only spirally bending deformation achieved without intervention, sophisticated patterns would realize by elaborately preorganizing. As shown in Figure 3c, a series of attractive “plastic flowers” were designed. PDMS strips served as petal and hydrogel as the bud of flowers. Before dehydration, original petals were horizontal with center of flower poking to the tip of syringe needle. The factors which change the driving forces, like contact areas, geometrical shape, and relative arrangement, all played essential roles in determining the eventual morphologies of flowers. The bigger size of bud which meant greater driving forces, the higher bending curvature of petal, thus sample c′ presented the smallest bending curvature in vertical. And, different geometrical shapes of hydrogel could result in more than one bending orientations. For example, comparing sample a′ and b′ in Figure 3c, there also appeared horizontal spiral^29^ in sample a′ except for bending vertically, and sample b′ with isosceles triangle bud displayed axial bending at the tip of petal. Additionally, to get flower with uniform bending curvatures, programing mini‐gel strips on PDMS petal regularly was useful, like Figure 3c′,d′, bending curvatures in four arms of petal were more uniform than other patterns. The other flowers we had fabricated are exhibited in Figure S7 (Supporting Information). It could be reasonably deduced that infinite patterns should be precisely designed beyond the configurations demonstrated above.

Taking advantage of responsiveness to Ca^2+^ which could interact with vacant guluronic acid (G unit) of alginate moieties, shape memory of hydrogel was explored. We regulated the shape of *i*‐PAP hydrogel through introducing or eliminating Ca^2+^ inside the gel. To conduct shape writing procedure, two samples of *i*‐PAP hydrogel with different thickness (thickness: 1.5/0.75 mm, width: 1 cm) were tailored and curled in spiral (type A, 1.5 mm thick) or annular cylindrical (type B, 0.75 mm thick) on glass rods. The thinner (0.75 mm) film sample was enwound in annular cylinder layer‐by‐layer to better support its own weight since it was much softer than thicker gels. With ionic bonding between Ca^2+^ and alginate‐PBA as the reversible crosslinks, this temporary shape could be recorded by immersing hydrogel in 0.1 mol L^‐1^ CaCl_2_ solution and easily eliminated by being transferred into EDTA solution (**Figure** **4**a). Shape programing and immersing procedures made the straight samples shape morphed, and helical shape or annular cylinder was memorized even assisting glass rod was released. Preprogrammed *i*‐PAP hydrogel maintained a stable helical configuration when put into CaCl_2_ solution (see Video S1, Supporting Information). Moreover, when resoaked in the solution of CaCl_2_, decalcified hydrogel was capable of morphing into spiral shape (Figure S8, Supporting Information). Interestingly, Na_2_CO_3_ basic solution was beneficial to weaken the temporary shape of hydrogel as well (Figure S9, Supporting Information). The constructed helical hydrogel recovered to its original state because of the releasement of calcium ions.

**Figure 4:**
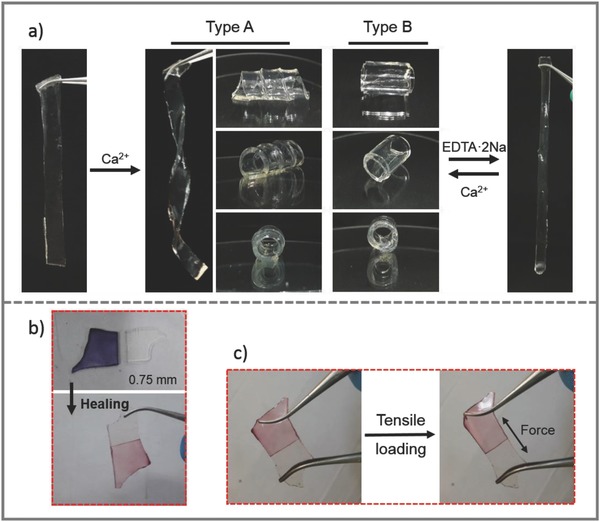
Shape memory and self‐healing properties of *i*‐PAP hydrogels respond to external Ca^2+^ and alkaline conditions, respectively. a) Reversible shape memory effect occurs in the presence of Ca^2+^ and could be erased by immersing in EDTA·2Na solution. b) Hydrogel images of before and after healing. c) Loading the healing hydrogel with biaxial tensile force for several cycles, the gel still maintains an intact.

In spite that shape memory and self‐healing are two kinds of contradictory properties,^30^ automatic self‐healing ability of *i*‐PAP hydrogel in alkaline conditions was achieved. As have been extensively studied, boronic acid groups have high binding affinity with diols when pH of surroundings is above the p*K*a of boronic acid, forming dynamic boronate esters.^31^ Considering that phenylboronic acid and diol units coexisted in the gel, self‐healing between cut surfaces of *i*‐PAP hydrogel was expected. Before cut into two halves, hydrogel sample was immersed in NaOH for a few seconds to offer a suitable alkaline condition for generating diol–boronate anion complex. The incisions of the cut halves were put together and contacted with each other. Not surprisingly, automatic self‐healing at 4 °C was observed after a week (Figure 4b). In spite of being loaded to biaxial tensile force, the healing gel resulting from dynamic boronate ester bonds was strong enough to withstand several tensile cycles without rupture (Figure 4c and Video S2, Supporting Information). The stress–strain curve of healed gel coincided partially with result of virgin alkaline‐conditioned gel and the joint reformed between the cut surfaces could resist a fracture stress up to 4.0 kPa (Figure S10, Supporting Information). The *i*‐PAP hydrogel treated with NaOH possesses better recovery and self‐healing property and is more preferable substitute material demanding for antivulnerable and repeatable.

In conclusion, this work successfully provided a smart hydrogel with multiple functionalities and stretchability. This multiresponsive hydrogel showed responsiveness to three categories of ordinary stimuli, namely, moisture, multivalent cations, and pH, exhibiting amusing actuation function, excellent shape memory, and automatic self‐healing properties in low temperature. Based on the mismatch of volume change ability after dehydration, we fabricated a series of “plastic flowers” with various patterns by assembling hydrogel strips with hydrophilic PDMS films to validate actuation function of hydrogel. In theory, appearance of flower could be elaborately regulated by tuning size, geometry, and distribution of hydrogel sample. With compatible multifunctionality and stretchability, this smart hydrogel broadens the scope of functional materials and improves the flexibility and versatility of hydrogel applications, which have great prospects to be used in fields of actuators, soft robotics, electronic skin, and other applications.

## Conflict of Interest

The authors declare no conflict of interest.

## Supporting information

Supplementary

Supplementary

Supplementary
