# Supplementary material for: Stretchable Multiresponsive Hydrogel with Actuatable, Shape Memory, and Self‐Healing Properties
Source: Adv Sci (Weinh). 2018 Jun 10;5(8):1800450. doi: 10.1002/advs.201800450 (PMC6096994; doi:10.1002/advs.201800450)
Supplement: Supplementary file 1 — Supplementary [file ADVS-5-1800450-s002.pdf]

Copyright WILEY-VCH Verlag GmbH & Co. KGaA, 69469 Weinheim, Germany, 2018.

ADVANCED SCIENCE Open Access

Supporting Information

for *Adv. Sci.,* DOI: 10.1002/advs.201800450

Stretchable Multiresponsive Hydrogel with Actuatable, Shape Memory, and Self-Healing Properties

*Feng Zhang, Ligui Xiong, Yongjian Ai, Zhe Liang, and Qionglin Liang**

Copyright WILEY-VCH Verlag GmbH & Co. KGaA, 69469 Weinheim, Germany, 2018.

Supporting Information

Stretchable Multi-responsive Hydrogel with Actuatable, Shape memory and Self-healing Properties

*Feng Zhang[&], Ligui Xiong[&], Yongjian Ai, Zhe Liang, and Qionglin Liang**

1. Preparation of *i*-PAP hydrogels.

We synthesized *i*-PAP hydrogel by a one-pot method. Before the hydrogel fabrication, we firstly prepared the PBA grafted alginate polymer (Alginate-PBA) according to a previously reported method [1] to get the lyophilized Alginate-PBA polymer. Then, acrylamide monomer and Alginate-PBA (the weight ratio of acrylamide to Alginate-PBA was 8:1 and the total solid content was 14 wt %) were dissolved in deionized water. To make sure Alginate-PBA completely dissolved in water, degassing in a vacuum chamber is operated. Subsequently, we added 0.00154 g/mL N,N'-methylenebisacrylamide (MBAA) as the crosslinking agent, 0.05 g/mL ammonium persulphate as a thermo-initiator and 0.775 g/mL N,N,N',N'-tetramethylethylenediamine as an accelerator for polyacrylamide. The amounts of MBAA, APS and TEMED are 0.028 mol%, 0.031 mol% and 0.152 mol%, respectively, molar ratio relative to acrylamide monomer.[2] Next, the mixture solution was transferred into a glass mould (75.0 mm×50.0 mm× 0.75 mm or 75.0 mm×50.0 mm×1.5 mm) to fabricate the hydrogel film or into a hollow glass tube (Diameter:1 mm, Length: 100 mm) with one end sealing to get the hydrogel fiber. After that, the molds were heated in an oven at 50℃ for 5h. Finally, we got synthesized *i*-PAP hydrogel by removing the gel from glass mould carefully. The prepared *i*-PAP hydrogels were stored at 4 ℃ to prevent dehydration.

The Ca-conditioned hydrogels and alkaline-conditioned hydrogels were obtained by immersing tailored *i*-PAP hydrogel into $CaCl_2$ and 1 mol/L NaOH solutions respectively. The principle of condtiitoned hydrogels formation are illustrated in Figure S1. The soaking time and concentration of treating solution are changeable depending on experimental requirements.

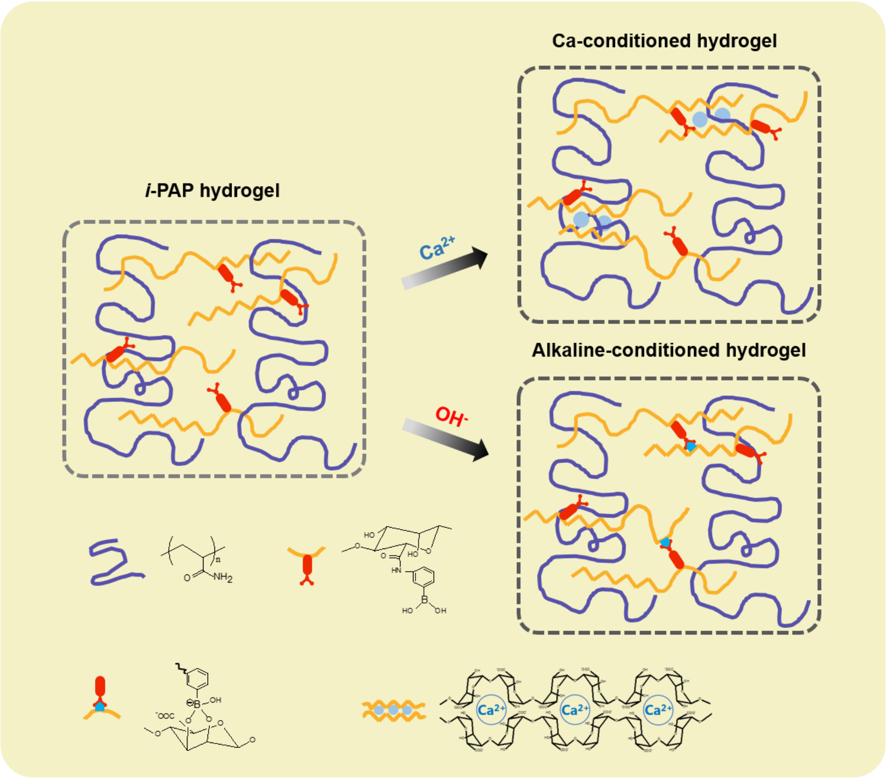

Figure S1. Schematic to illustrate the formation of Ca-conditioned and alkaline-conditioned hydrogels.

2. Characterizations

We investigated the micro-structure, possible crosslinking and mechanical property of *i*-PAP control hydrogel and conditioned hydrogels by SEM (scanning electron microscopy), FTIR (Fourier transform infrared spectroscopy), and rotational rheometer. These results are shown in Figure S2-S4. Unless otherwise stated, the treating condition of Ca-conditioned

hydrogel was fixed at 0.1 mol/L $CaCl_2$ solution for 3h and alkaline-conditioned hydrogel at 1 mol/L NaOH solution for 1min among all the characterizations.

2.1 SEM

SEM samples of hydrogel were prepared by firstly pre-freezing hydrogels at -20 ℃ followed with lyophilizing in a vacuum freeze dryer. The SEM images of Ca-conditioned hydrogel and alkaline-conditioned hydrogel were shown in Figure S2. Comparing with control *i*-PAP sample, conditioned hydrogels showed compact and smooth internal structure, rather than porosity, indicating that introducing Ca^{2+} or supporting with an alkaline condition transformed the bonding interaction and finally resulted in microstructure alteration. Even so, internal structure of alkaline-conditioned hydrogel was not as compact as Ca-conditioned sample with rugose surface.

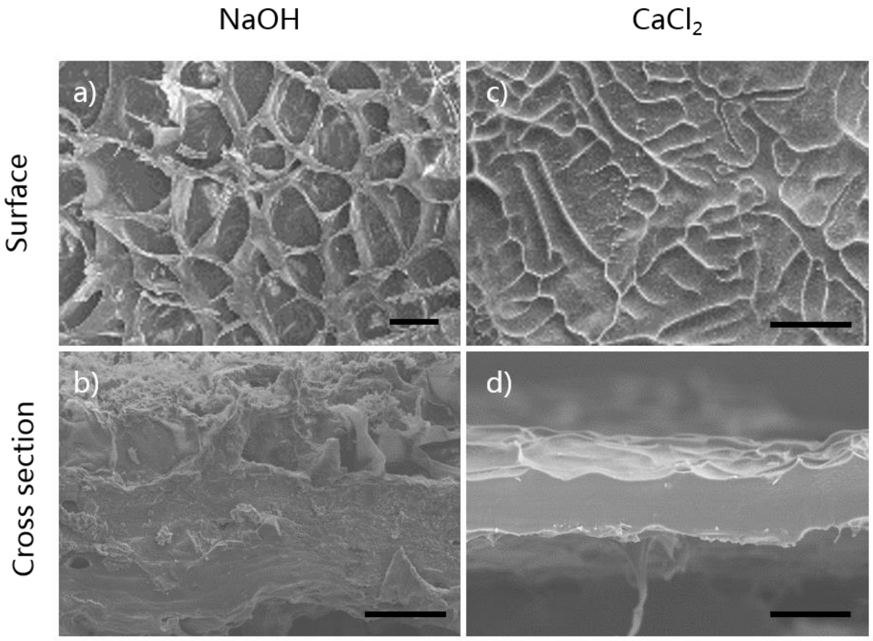

Figure S2. SEM images of lyophilized alkaline-conditioned and Ca-conditioned hydrogels. The treating time of $CaCl_2$ was 3h, while NaOH was 1min. All the scale bars are 50μm.

2.2 FTIR

To analyze possible crosslinking in hydrogel after immersing with $CaCl_2$ or NaOH solution, we carried out Fourier Transform Infrared (FTIR) spectra of control *i*-PAP gel, Ca-conditioned gel, and alkaline-conditioned gel (Figure S3). FTIR samples were prepared as same as SEM sample. The peaks at 3320 cm^{-1} and 3180 cm^{-1} attributed to the stretching vibration of C-OH and N-H, respectivily. The band 1650 cm^{-1} was corresponding to asymmetric COO^- stretching in alginate moieties, and band at 1610 cm^{-1} was related to N-H deformation for primary amine in polyacrylamide chains.[3] Analyzing the spectra of control and Ca-conditioned samples, there was no siginificant shifting peak, this results could be explained that Ca^{2+} replaced Na^+ without new covalent bond formation. Besides, in the spectra of alkaline-conditioned sample, the emerging of siginificant absorption band (1420 cm^{-1}) which attributed to C-B vabriation implied the instability of C-O-B bonds.[4]

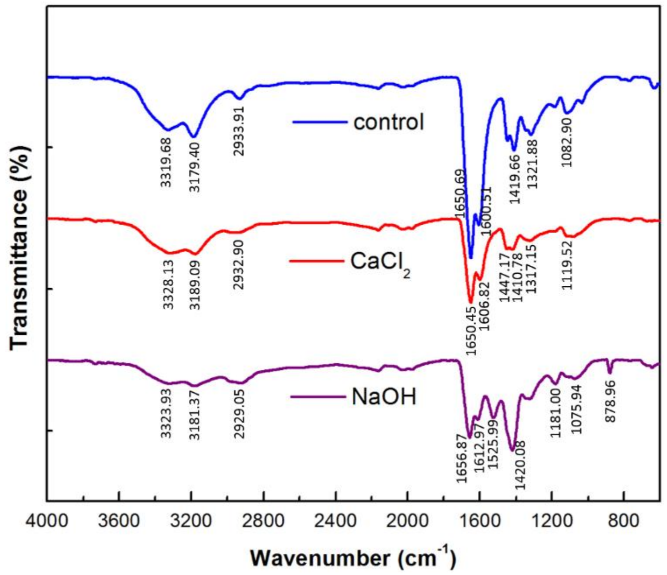

Figure S3. FTIR spectra of control *i*-PAP hydrogel, Ca-conditioned hydrogel and alkaline-conditioned hydrogel.

2.4 Mechanical performance

Mechanical performances of control, Ca-conditioned and alkaline-conditioned *i*-PAP hydrogels were determined by AR-G2 rheometer using ϕ8 mm parallel-plate as the roator. Rotation frequency-sweep tests at 1% shear strain were carried out over the frequency range 0.01-10 Hz at room temperature. The storage modulus E' and loss modulus E'' for control, Ca-conditioned and alkaline-condtitioned gels were tested as the frequency changes (Figure S4). As expected, Ca-conditioned hydrogel showed greater mechanical strength than control and OH-conditioned samples, due to the ionic crosslinking formationdue between Ca^{2+} and vacant carboxylic groups in alginate.

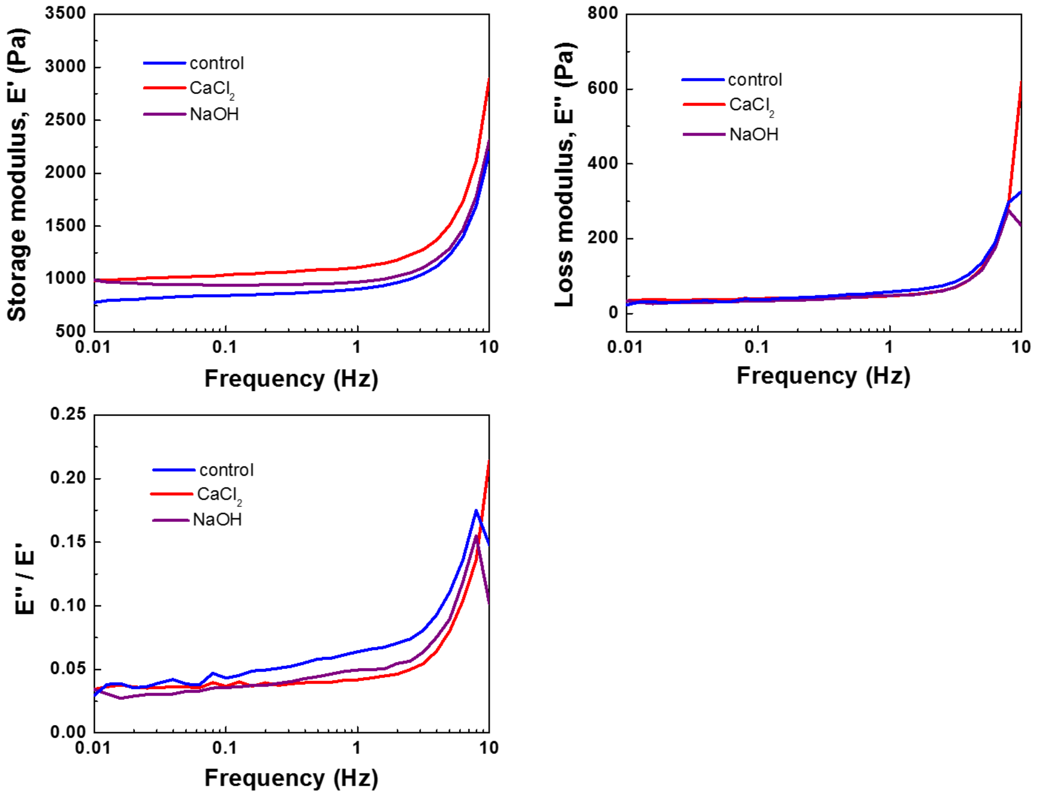

Figure S4. Mechanical performances of *i*-PAP hydrogel, Ca-conditioned hydrogel, and alkaline-conditioned hydrogel. a), Storage modulus E'. b) Loss modulus E''. c) The ratio between E'' and E'.

Tensile stress-strain curves and mechanical strength of PAM gel and Alginate-PBA gel were also provieded (Figure S5). Although Alginate-PBA could be gelated in two different

ways, namely multications and basic pH, the Alginate-PBA gel crosslinked in basic solutions was unable to form uniform film. Thus, stress-strain curve of Alginate-PBA/OH gel was not shown here.

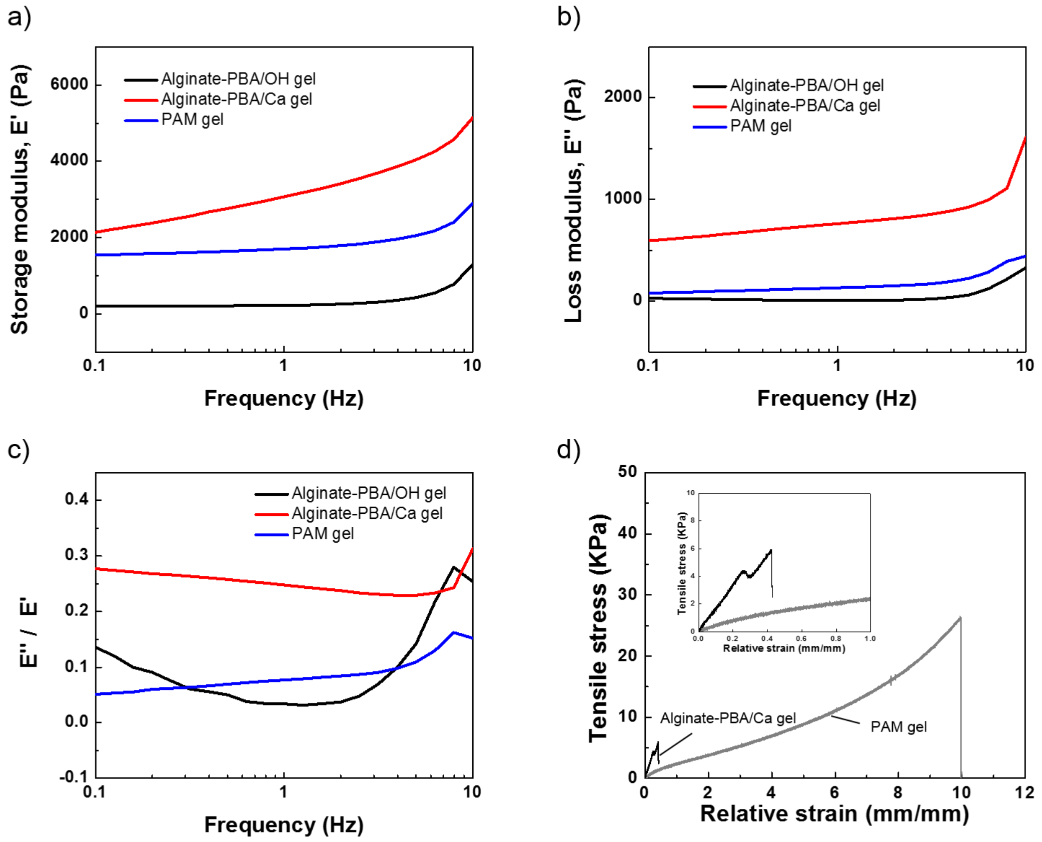

Figure S5. Mechanical performances of pure PAM hydrogel, Alginate-PBA/Ca hydrogel, and Alginate-PBA/OH hydrogel. a) Storage modulus E'. b) Loss modulus E''. c) The ratio between E'' and E'. d) Tensile stress-relative strain curves of PAM hydrogel and Alginate-PBA/Ca hydrogel.

3. Home-made device for stretchability study

To compare the stretchability of *i*-PAP hydrogel and other two conditioned hydrogels, we built up a simply devices based on syringe pump (Figure S6). The syringe pump with adjustable injection rate supported an uniform tensile to hydrogel sample. During the stretching experiments, the size of all hydrogel samples were 5mm*5mm*0.75mm and tensile rate was kept 100% strain /min. Considering the property of hydrogel samples, the most

challenge of our home-made device was to immobilize two ends of hydrogel. Through a multitude of trials, we finally selected two layers of filter paper with sample forming sandwich-structure to fix two ends of samples and the filter paper of sandwich-structure was glued by electrical tape. After preparing tensile sample, we seperately glued the two end of sample to the fixed station and moiving station of syringe pump using Very High Bond (VHB) 4905 tape and then loaded with 200g weights on two ends.The potography of sample stretching using home-made device was demonstrated in Figure S6b.

Once the sample was fixed in the device, we measured and recorded the original length (L_0) of hydrogel sample. Stretching sample at 100% strain/min rate and monitiring the total length (L) real time unitil it achieved critical stretching length (L_m). Critical tensile situation was defined as the moment that hydrogel was just rupturing. The whole procedures was schamically illustated in Figure S6a. Displacement (S), representing the changing length of sample during stretching, was also known as the strain.

In our work, relative strain was the ratio of strain to its original length, the formula can be written as equation (1)：

$$Relative\ strain = \frac{S}{L_0} = \frac{L - L_0}{L_0} \tag{1}$$

The relative critical strain was determined as equation (2)：

$$Relative\ critical\ strain = \frac{S_m}{L_0} = \frac{L_m - L_0}{L_0} \tag{2}$$

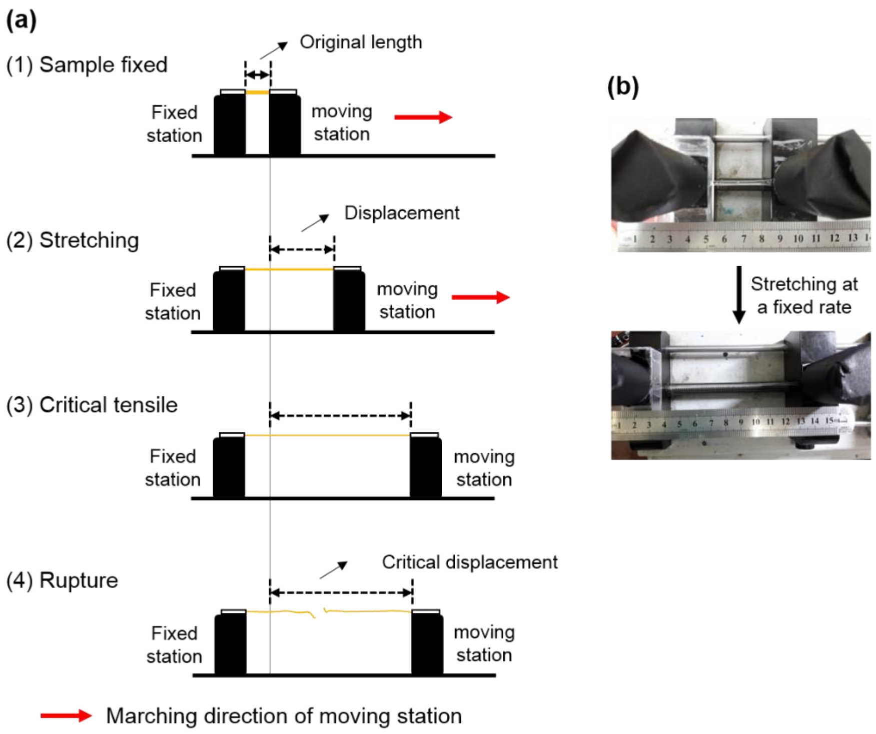

Figure S6. Home-made device for study the stretchability performance of hydrogel samples. a) Schematic procedures of tensile experiment; b) Photography of a hydrogel sample stretching at a fixed rate by the home-made devices.

4. PDMS film fabrication and hydrophilic modification

The polydimethylsiloxane (PDMS) film was prepared follwing a previously reported protocol[5] with a minor modification. In brief, the PDMS precursor mixed by polydimethylsiloxane base and curing agent with a weight ratio of 10:1 was firstly degassed under vacuum for 30 min. Then, spinning the degassed PDMS precursor onto a silanized silicon wafer at a speed of 400 rpm for 6s. Sequentially, heating the wafer for 5 min at 90 °C to obtain a curing PDMS film with height of 500 μm. Owing to the different wettability of hydrogel and PDMS film, the hydrogel would detach from the hydrophobic PDMS film when volume shrunk.

To transform the original hydrophobic of PDMS surface to hydrogphilic and support the affinity to hydrogel, we cured the PDMS surface in a commeicial oxygen plasma cleaner for 30 s to get a temporary hydrophilic film.

5. Demonstration of actuatable functionality

There were other patterns of plastic flower regulated, as shown in Figure S7. In Figure S7a, we selected four homogeneous *i*-PAP hydrogel fibers with diameter of 1.1 mm to be distributed on the crosssection of two PDMS strips. The contact region between hydrogel sample and PDMS film was a line (1-dimension), while in Figure S7b-d, the contact region was a rectangle or triangle (2-dimension) – the geometry of hydrogel sample. The differences of contact resgion resulted in diverse patterns of "plastic flower".

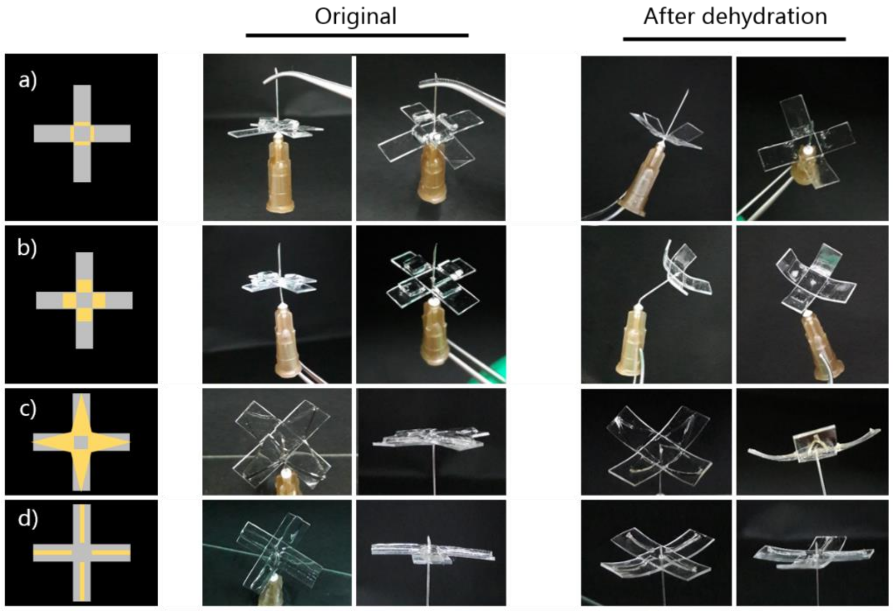

Figure S7. Supplementary examples of different patterns of "plastic flower".

6. Demonstration of shape memory

Decalcified hydrogel was re-soaked in the solution of $CaCl_2$ to further demonstrate the reversible shape memory feature of *i*-PAP hydrogel (Figure S8).

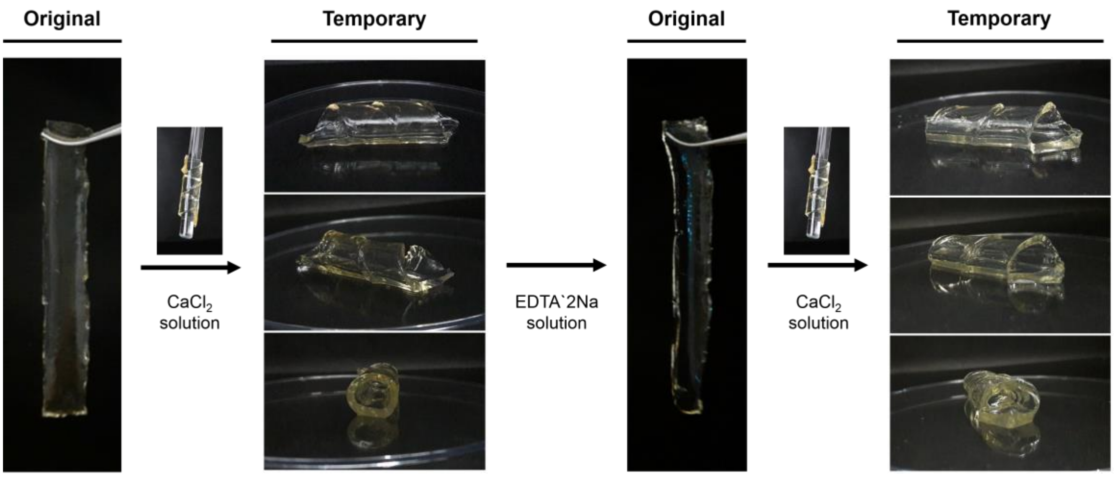

Figure S8. Reversible shape memory capability of *i*-PAP hydrogel.

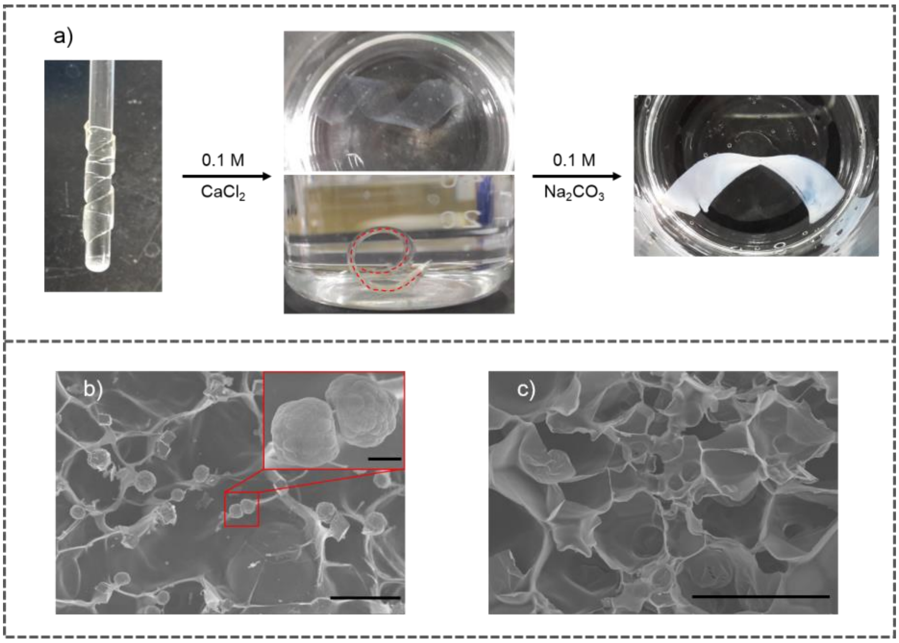

Figure S9. a) Performance of hydrogel successively soaked in Ca^{2+} ion and Na_2CO_3 solutions. Surface (b) and cross-section (c) scanning electron microscope images of hydrogel successively immersed into $CaCl_2$ and Na_2CO_3 solutions in both. Scale bars in b and c are 50 μm with 5 μm in the insert.

Besides, how the hydrogel performs in a solution with both Ca^{2+} and basic pH was illustrated and results were diaplayed in Figure S9. Considering the low solubility of

$Ca(OH)_2$,we adopted a two-step experiment by putting a hydrogel in Ca^{2+} solution and then being transferred into Na_2CO_3 (0.1M) solution. As shown in Figure S9a, the spiral shape could be kept in Ca^{2+} solution. While being transferred into Na_2CO_3 solution, the hydrogel gradually despiralized with increasing thread pitch because the Ca^{2+} was combined with carbonate ion forming $CaCO_3$. Also, the precipitation of $CaCO_3$ only distributed on the surface of hydrogel rather than in the internal structure (figure S9b-c).

7. Demonstration of self-healing

The tensile ability of healed sample was also measured by a loading tester and the results were showing in Figure S10. The curve of healed sample coincided with result of virgin alkaline-conditioned gel, which indicated that there was no loss of mechanical strength in healed gel. And it could also stretched more than 2 times to its origianl length with a fracture stress up to 4.0 KPa.

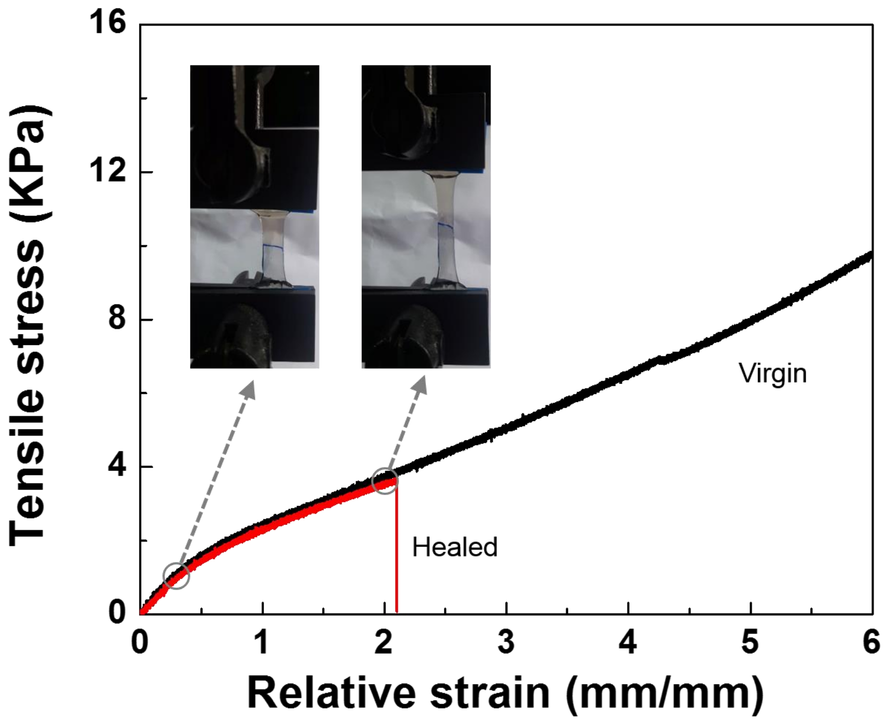

Figure S10. Tensile stress-relative strain curves of virgin alkaline-conditioned and healed sample.

8. Supplementary Videos

Video S1 *i*-PAP hydrogel preprogramming with spiral shape maintained a stable temporary configuration in response to Ca^{2+} stimuli.

Video S2 Loading the healing hydrogel sample with biaxial tensile force for several cycles.

9. References

[1] A. Pettignano, S. Grijalvo, M. Haring, R. Eritja, N. Tanchoux, F. Quignard, D. Diaz Diaz, *Chem Commun (Camb)* **2017**, *53*, 3350-3353.

[2] C. H. Yang, M. X. Wang, H. Haider, J. H. Yang, J. Y. Sun, Y. M. Chen, J. Zhou, Z. Suo, *ACS Appl Mater Interfaces* **2013**, *5*, 10418-10422.

[3] J. Y. Sun, X. Zhao, W. R. Illeperuma, O. Chaudhuri, K. H. Oh, D. J. Mooney, J. J. Vlassak, Z. Suo, *Nature* **2012**, *489*, 133-136.

[4] Z. Sebestyen, K. Mathe, A. Buvari-Barcza, E. Vass, F. Ruff, J. Szeman, L. Barcza, *Carbohydr Res* **2011**, *346*, 833-838.

[5] Y. Li, L. Li, Z. Liu, M. Ding, G. Luo, Q. Liang, *Microfluid Nanofluid* **2016**, *20*.
